# Different Modulatory Effects of Four Methicillin-Resistant *Staphylococcus aureus* Clones on MG-63 Osteoblast-Like Cells

**DOI:** 10.3390/biom11010072

**Published:** 2021-01-07

**Authors:** Nicolò Musso, Giuseppe Caruso, Dafne Bongiorno, Margherita Grasso, Dalida A. Bivona, Floriana Campanile, Filippo Caraci, Stefania Stefani

**Affiliations:** 1 Department of Biomedical and Biotechnological Sciences (BIOMETEC), University of Catania, 95125 Catania, Italy; nmusso@unict.it (N.M.); dbongio@unict.it (D.B.); dalidabivona@gmail.com (D.A.B.); f.campanile@unict.it (F.C.); stefanis@unict.it (S.S.); 2 Oasi Research Institute-IRCCS, Via Conte Ruggero, 73, 94018 Troina, Italy; forgiuseppecaruso@gmail.com (G.C.); grassomargherita940@gmail.com (M.G.); 3 Department of Drug Sciences, University of Catania, 95125 Catania, Italy

**Keywords:** *Staphylococcus aureus*, osteoblast-like cells, internalization, inflammation, immune system, eukaryotic host­–pathogen interaction, cytokines

## Abstract

*Staphylococcus aureus* is a Gram-positive bacterium responsible for a variety of mild to life-threatening infections including bone infections such as osteomyelitis. This bacterium is able to invade and persist within non-professional phagocytic cells such as osteoblasts. In the present study, four different *S. aureus* strains, namely, 2SA-ST239-III (ST239), 5SA-ST5-II (ST5), 10SA-ST228-I (ST228), and 14SA-ST22-IVh (ST22), were tested for their ability to modulate cell viability in MG-63 osteoblast-like cells following successful invasion and persistence. Methicillin-sensitive *S. aureus* (MSSA) ATCC-12598-ST30 (ST30) was used as control strain. Despite being proven that ST30, ST239, and ST22 have a similar ability to internalize and persist in MG-63 osteoblast-like cells under our experimental conditions, we demonstrated that the observed decrease in cell viability was due to the different behavior of the considered strains, rather than the number of intracellular bacteria. We focused our attention on different biochemical cell functions related to inflammation, cell metabolism, and oxidative stress during osteoblast infections. We were able to show the following: (1) ST30 and ST239 were the only two clones able to persist and maintain their number in the hostile environment of the cell during the entire period of infection; (2) ST239 was the only clone able to significantly increase gene expression (3 and 24 h post-infection (p.i.)) and protein secretion (24 h p.i.) of both interleukin-6 (IL-6) and tumor necrosis factor alpha (TNF-α) in MG-63 osteoblast-like cells; (3) the same clone determined a significant up-regulation of the transforming growth factorbeta 1 (TGF-β1) and of the metabolic marker glyceraldehyde 3-phosphate dehydrogenase (GAPDH) mRNAs at 24 h p.i.; and (4) neither the MSSA nor the four methicillin-resistant *S. aureus* (MRSA) strains induced oxidative stress phenomena in MG-63 cells, although a high degree of variability was observed for the different clones with regard to the expression pattern of nuclear factor E2-related factor 2 (Nrf2) and its downstream gene heme oxygenase 1 (HO-1) activation. Our results may pave the way for an approach to *S. aureus*-induced damage, moving towards individualized therapeutic strategies that take into account the differences between MSSA and MRSA as well as the distinctive features of the different clones. This approach is based on a change of paradigm in antibiotic therapy involving a case-based use of molecules able to counteract pro-inflammatory cytokines activity such as selective cytokine signaling inhibitors (IL-6, TNF-α).

## 1. Introduction

Some pathogens are able to internalize and establish persistent and lifelong infections. Several of these pathogens manage to evade the host immune system and cause disease by replicating inside the host cells [1,2]. The main characteristic of bacterial strains is “to adapt the metabolism”, so as to escape the defenses of the cell for a longer or shorter period. However, this system is not able to completely eliminate the eukaryotic cell response, which differs according to the type of offense [1].

Over the past fifty years, *Staphylococcus aureus*, representing one of the main adaptable human pathogens and responsible for community and nosocomial infections, has been able to acquire numerous resistance and virulence genes [3]. Furthermore, this bacterium has shown the ability to invade and persist within non-professional phagocytic cells such as osteoblasts, to remain alive in the intracellular environment, and to evade the immune system (the so-called “phagosomal escape”) [4]. This intracellular persistence has often been associated with a metabolic variant, known as small colony variant (SCV), able to persist inside human cells and refractory to antibiotic therapy [5].

Orthopedic infections, such as osteomyelitis and prosthetic joint infections, are recurrent and chronic infections often requiring prolonged antimicrobial therapies and surgical interventions owing to the challenges in eradicating the bacteria from osteoblasts [6,7]. The prevalence of methicillin-sensitive *S. aureus* (MSSA) in this infection is higher than methicillin-resistant *S. aureus* (MRSA), but these pathogens are often associated with antimicrobial resistance, with high rates of patient hospitalization and mortality [8]. A recent study reported that some clones belonging to ST239, ST642, and ST107 are more commonly associated with orthopedic infections [9]. Among the clones of healthcare-associated MRSA (HA-MRSA), mainly spread in Italy, we found ST239-SCC*mec*III (ST239), ST5-SCC*mec*II (ST5), ST228-SCC*mec*I (ST228), and ST22-SCC*mec*IV (ST22) [10]. ST5 or the New York/Japan strain is one of the most widely spread HA-MRSA strains, particularly in the USA, Japan, Canada, South Korea, Australia, and Europe [11,12], while ST228, known as the German or Italian clone, has been predominant in Italy for more than 10 years. This clone was associated in particular with bacteremia, endocarditis, and low respiratory tract infections [10,13]. The gentamicin-susceptible clone ST22 was recognized as the most successful and rapidly disseminating HA-MRSA clone first in England in 1990, then throughout Europe. This clone was also able to cause outbreaks in the community, and replaced other clones in New Zealand, Australia, and India. In Italy, it replaced the most widespread clone ST228 [12,14].

The activation of the immune system and inflammatory processes are relevant factors in many pathologies such as diabetes [15], cancer [16], systemic [17], and neurodegenerative diseases [18,19]. Furthermore, inflammation, characterized by pro-inflammatory cytokines’ up-regulation, plays a critical role during the onset and progression of bacterial infections [20,21]. In particular, *S. aureus*-induced infection determines a deregulated production of pro-inflammatory cytokines, including tumor necrosis factor alpha (TNF-α) and interleukin-6 (IL-6), as well as of several chemokines [22,23]. The deregulated production of these cytokines is also correlated to oxidative stress, a condition characterized by an imbalance between pro-oxidant (excess) and antioxidant (deficiency) species [24,25]. Cells are able to activate the antioxidant machinery to maintain homeostasis, including the activation of nuclear factor E2-related factor 2 (Nrf2), regulating several hundred genes involved in the antioxidant defense response [26].

Among the different types of cells that are infected by *S. aureus*, osteoblasts represent one of the most studied. Once infected, these cells participate in the initiation as well as maintenance of the inflammatory process through the production of cytokines and the recruitment of immune cells to the inflammation site [6]. Recently, Horn et al. discussed the ability of *S. aureus* to internalize in phagocytic (macrophages) and non-phagocytic (osteoblasts) cells, highlighting how bacterial intracellular persistence is connected to immune evasion phenomena and chronic infection [27]. Particularly in the case of osteomyelitis, *S. aureus* infection of osteoblasts has been linked to the overproduction of pro-inflammatory markers, decreased cell activity, and increased cell death through induction of apoptosis-dependent and independent mechanisms; this bacterial infection was also responsible for the deregulated homeostasis between osteoblasts and osteoclasts in osteomyelitis [28,29].

In the present study, we investigated the intracellular persistence and toxicity of four genetically different strains of MRSA in MG-63 osteoblast-like cells. In order to shed more light on the different behavior observed in these strains, we also determined their influence on the gene expression of IL-6, TNF-α, transforming growth factor beta 1 (TGF-β1), and glyceraldehyde 3-phosphate dehydrogenase (GAPDH); of Nrf2 and its downstream effector heme oxygenase 1 (HO-1); as well as of inducible nitric oxide synthase (iNOS) and NADPH oxidase 2 (Nox-2). The human MG-63 osteoblast-like cell line was selected not only because it represents a validated model to study the pro-inflammatory response to bacterial infection [30,31,32], but also because it shows a number of features typical of an undifferentiated osteoblast phenotype. Also noticeable and worthy of note are the synthesis of collagen types I and III; the low basal expression of alkaline phosphatase, which is increased following administration of 1,25-dihydroxyvitamin D (1,25(OH)2D); and the production of osteocalcin in the presence of 1,25(OH)2D [33,34,35].

## 2. Materials and Methods

### 2.1. Materials and Reagents

Materials and reagents were all of analytical grade and were purchased from Sigma (St. Louis, MO, USA) or Thermo Fisher Scientific Inc. (Pittsburgh, PA, USA), unless otherwise specified. *Staphylococcus aureus* subsp. aureus Rosenbach (ATCC^®^ 12598™) (Cowan ST30-t076) and the human osteosarcoma cell line MG-63 (ATCC^®^ CRL-1427™) were purchased from American Type Culture Collection (ATCC, Manassas, VA, USA). C-Chip disposable hemocytometers were obtained from Bulldog Bio, Inc. (Portsmouth, NH, USA). QuantiTect SYBR Green PCR Kit, RNA extraction kit (RNeasy Mini Kit), RNase-free DNase Set, QuantiTect Primers, and Custom Multi-Analyte ELISArray Kit were all purchased from Qiagen (Hilden, Germany). Eppendorf LoBind Microcentrifuge Tubes PCR Clean (1.5 mL) and PCR tubes were supplied by Eppendorf (Hamburg, Germany).

### 2.2. Bacterial Strains

For this study, four different bacterial strains, belonging to two diverse STs, namely, 2SA-ST239-III (ST239), 5SA-ST5-II (ST5), 10SA-ST228-I (ST228), and 14SA-ST22-IVh (ST22), were used [12]. All MRSA strains used in this study were clinical isolates selected from a large collection of MRSA strains isolated during an Italian national survey conducted in 2012. The strains were phenotypically and molecularly characterized as previously reported [10,36]. These strains had already been studied for their ability to internalize and persist in MG-63 osteoblast-like cells [37]. The MSSA ATCC-12598-ST30 (Cowan ST30-t076; ST30), an invasive isolate, was used as the control strain for invasion and persistence assays, *imaging flow cytometry, as well as* gene and protein expression analysis [38]. Table 1 reports the phenotypical and molecular characteristics of the bacteria included in this study.

### 2.3. Eukaryotic Cell Culture Preparation

Infection experiments were performed on the human osteosarcoma cell line MG-63. During the expansion period, the cells were grown in 75 cm^2^ flasks with modified Eagle’s medium (MEM), supplemented with 10% fetal bovine serum (FBS), 1X GlutaMAX™, and 100 U/mL of penicillin/streptomycin solution. Cells were incubated at 37 °C in a humidified atmosphere with 5% CO_2_/95% air and the medium was changed twice weekly. Twenty-four hours prior to infection, cells were harvested, counted with a C-Chip disposable hemocytometer, and seeded (full medium in absence of antibiotics) in 6- or 96-well plates at the appropriate density.

### 2.4. Infection of MG-63 Cells

The intracellular frequency of the five different bacterial strains was evaluated in MG-63 osteoblast-like cells at a multiplicity of infection (MOI) of 100:1. This MOI was selected based on previous experiments in which MG-63 cells were infected with the ST30 clone, our control strain, at an increasing MOI (12, 50, 100, and 200), observing that, with an MOI of 12 or 50 the ability of *S. aureus* to internalize in MG-63 cells was very low, whereas an MOI of 200 was quite cytotoxic. Our selection is also supported by many publications where the MOI of 100:1 was reported as the standard for osteoblast infections [39,40,41,42,43]. Bacterial isolates were grown in brain heart infusion (BHI) broth at 37 °C overnight. The bacterial concentration was evaluated by optical density at 600 nm using the GENESYS™ 10S UV-Vis Spectrophotometer (Thermo Fisher Scientific, Waltham, MA, USA). Bacterial suspensions were prepared using MEM supplemented with 1X GlutaMAX™ and 10% FBS in the absence of penicillin/streptomycin. The same medium composition was also used to grow the MG-63 cells, which were infected for 3 and 24 h in antibiotic-free conditions. Extracellular bacterial lysis was carried out for 1 h at 37 °C using 100 mg/mL of lysostaphin [37].

### 2.5. Evaluation of the Frequency of Internalization and Intracellular Persistence by Colony-Forming Units (CFUs) and Spot Counting

The amount of intracellular bacteria at 3 and 24 h post-infection (p.i.) was estimated by CFUs/mL counting, as previously described [40]. In order to estimate the percentage of bacteria internalized in MG-63 cells, the following factors were considered: (1) MOI; (2) number of cells; and (3) number of CFUs counted after cell lysis.

The number of spots (bacteria) 24 h p.i. was measured by imaging flow cytometry, as previously described [40]. A representative sample of 10,000 events was acquired for each experimental condition.

### 2.6. Evaluation of Cell Viability by MTT Assay

To evaluate the effect of *S. aureus* internalization and persistence on the viability of MG-63 cells plated in 96-well plates (2.5 × 10^3^ cells/well) under our different experimental conditions, an MTT ([3-(4,5-dimethylthiazol-2-yl)-2,5-diphenyltetrazolium bromide]) assay was performed as previously described [44,45], with slight modifications. Two hours p.i., the extracellular bacterial lysis was obtained using 100 mg/mL of lysostaphin. After incubating for 1 h, the medium containing lysostaphin and lysed bacteria was discarded from each of the 96 wells. The cells were then washed twice with sterile phosphate-buffered saline (PBS) 0.01 M; 100 µL of MEM medium supplemented with 1X GlutaMAX™ and 10% FBS were added to each well. The MTT solution (20 µL at a concentration of 5 mg/mL) was added to each well 3 or 24 h p.i., followed by incubation (2 h) at 37 °C. At the end of the incubation step, the medium was removed and the formed formazan crystals were melted by adding 200 µL/well of anhydrous dimethyl sulfoxide (DMSO), with gentle stirring of the plate in a gyratory shaker for 10 min. As a final step, the 569 nm absorbance was read using a Synergy H1 Hybrid Multi-Mode Microplate Reader (Biotek, Shoreline, WA, USA).

We also performed a control experiment using five different concentrations of bacteria (~1300, ~3300, ~5400, ~21,000, and ~85,000 bacteria/well) under the same conditions employed for MG-63 cells. The aim of this experiment was to exclude any significant effect on the measured absorbance values resulting from bacterial activity. The results of this set of experiments showed that the bacteria, when used at concentrations ranging between ~1300 and ~3300, like in our experimental conditions, did not significantly contribute to the absorbance measured in the presence of MG-63 cells (Supplementary Figure S1).

### 2.7. Gene Expression Analysis by Quantitative Real-Time PCR (qRT-PCR)

The concentration of total RNA recovered from 3.5 × 10^5^ uninfected MG-63 cells (indicated as No Bacteria) or cells infected with ST30, ST239, ST5, ST228, or ST22 for 3 and 24 h was determined by measuring 260 nm absorbance with a NanoDrop^®^ ND-1000 (Thermo Fisher Scientific, Waltham, MA, USA). The RNA quality was tested by Qubit^®^ 3.0 Fluorometer (Thermo Fisher Scientific, Waltham, MA, USA). For reverse transcription, sample amplification, fluorescence data collection, and sample quantification, the same protocol was used as previously described [46,47]. The information of QuantiTect Primer Assays used for the gene expression analysis is reported in Table 2.

### 2.8. Cytokine Secretion Analysis by Enzyme-Linked Immunosorbent Assay (ELISA)

Quantification of IL-6, TNF-α, and TGF-β1 in cell culture supernatants from uninfected MG-63 cells or cells infected with ST30, ST239, or ST228 for 24 h was carried out using a Custom Multi-Analyte ELISArray Kit according to the manufacturer’s instructions [19].

### 2.9. Statistical Analysis

Statistical analysis was performed and the related graphs were prepared using the Graphpad Prism software, version 8 (Graphpad software, San Diego, CA, USA). Within-group comparison was performed by one-way analysis of variance (ANOVA) followed by Bonferroni multiple comparisons post hoc test. Only two-tailed *p*-values below 0.05 were considered as statistically significant. All experiments were performed at least in triplicate.

## 3. Results

### 3.1. The Intracellular Persistence and Number of Bacteria in MG-63 Osteoblast-Like Cells Vary Significantly among the Different Strains

**Figure 1:**
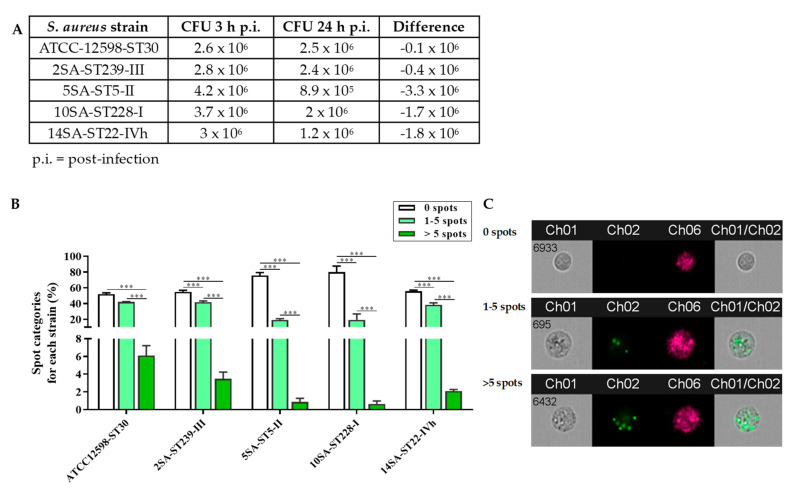
(**A**) Colony-forming units (CFUs)/mL counts measured following cell lysis for the five different *S. aureus* strains at 3 and 24 h post-infection (p.i.). (**B**) Percentage of spot categories (0 Scheme 1. to 5, and over 6 spots) for each strain at 24 h p.i. Values are reported as means ± SD of three independent experiments. Significant comparisons between each category are indicated by lines. *** Significantly different, *p* < 0.001. (**C**) Representative image showing MG-63 cells with no spots inside the cell (0 spots), a single MG-63 cells with three spots (1–5 spots), and a single MG-63 cell with 6 spots (>5 spots). CH1 = brightfield; CH2 = 505–560 nm; CH6 = side scatter (SSC) at 785 nm.

Figure 1A shows the amount of intracellular bacteria for the five different *S. aureus* strains considered, calculated as CFUs/mL, at 3 and 24 h p.i.

The CFUs/mL measured 3 h p.i. was quite similar for ST30, ST239, and ST22, ranging from 2.6 to 3 × 10^6^ CFUs/mL, while it was higher for ST5 (4.2 × 10^6^) and ST228 (3.7 × 10^6^) strains. The variation in the number of bacteria during the following 21 h, i.e., at 24 h p.i., was different for the five different *S. aureus* strains considered, as clearly shown by the differences in the CFUs/mL counts recorded at 3 and 24 h p.i. In fact, while in the case of ST30 and ST239, the measured CFUs/mL was pretty similar to that observed at 3 h p.i., the amount of bacteria measured at 24 h p.i. strongly decreased for ST5, ST228, and ST22.

To better examine the different ability to infect and persist among the five invasive *S. aureus* strains, the spot categories (0 spots, 1–5 spots, >5 spots) were calculated for each strain (%) (Figure 1B). Based on the average spot number per cell, a similar distribution of the three spot categories, i.e., % 0 spots > % 1–5 spots > % >5 spots (*p* < 0.001), was observed for almost all *S. aureus* strains considered. An exception was represented by ST30, for which the % 0 and % 1–5 spot categories were comparable. Supplementary Table S1 reports the existing interstrain differences obtained by comparing the percentages of the same spot category.

As clearly depicted in Figure 1, the internalization profiles of ST30, ST239, and ST22 were almost superimposable; in fact, there were no statistically significant differences for the three categories considered. However, a different behavior was observed for the remaining strains, ST5 and ST228, each of which showed significant differences for the 0 spots and 1–5 spots categories compared with the ST30, ST239, and ST22 strains. Of note, no significant interstrain differences were observed among the five bacterial strains for the >5 spots category.

### 3.2. Infection with ST30, ST239, ST5, ST228, or ST22 Strains Differently Affected MG-63 Osteoblast-Like Cell Viability

**Figure 2:**
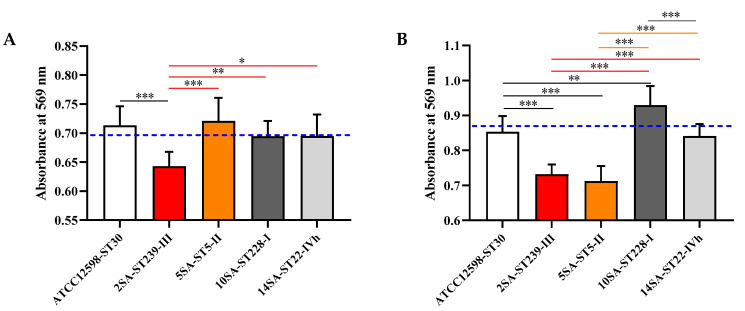
Changes in cell viability of MG-63 osteoblast-like cells infected at a multiplicity of infection (MOI) of 100:1 with five different *S. aureus* strains detected after (**A**) 3 h and (**B**) 24 h. Values are reported as absorbance measured at 569 nm and represent the means ± SD of at least five independent experiments. Blue-dotted line indicates the absorbance measured at 569 nm for uninfected MG-63 cells. Significant comparisons between each experimental condition are indicated by lines. * Significantly different, *p* < 0.05; ** significantly different, *p* < 0.01; *** significantly different, *p* < 0.001.

Figure 2 reports the changes in MG-63 cell viability after infection with the five different *S. aureus* strains at 3 and 24 h.

The viability of MG-63 cells infected with the ST239 MRSA strain, measured as absorbance at 569 nm, was significantly lower (*p* < 0.001) compared with that of cells infected with the reference control strain ST30 at 3 h p.i. (Figure 2A). The same applies for the comparison with the other MRSA strains (*p* < 0.001 vs. ST5; *p* < 0.01 vs. ST228; *p* < 0.05 vs. ST22) at 3 h p.i. The absorbance values measured for MG-63 cells infected with all other strains were very similar to those observed with the reference control strain at 3 h p.i. A quite different situation was observed 24 h p.i. In this case, both the ST239 and ST5 strains were able to decrease cell viability significantly compared with the ST30 (*p* < 0.001 for both) and with the ST228 or ST22 (*p* < 0.001) MRSA strains (Figure 2B). The infection of MG-63 cells with the other two strains yielded viability values comparable to (ST22) or even higher than (ST228; *p* < 0.01) those observed for cells infected with the reference strain (Figure 2B).

Overall, the results reported in Figure 2**,** along with previous studies [37], showed that the ability of *S. aureus* strains to infect and persist in MG-63 osteoblast-like cells is not always proportionally related to changes in cell viability. Therefore, the subsequent experiments aimed to determine whether bacteria internalization and the related changes in cell viability were associated with any variation in the different cellular biochemical functions, with inflammatory phenomena, and with the modulation of the antioxidant machinery.

### 3.3. The Longer Infection Time with ST239 Strain Leads to the Up-Regulation of IL-6, TNF-α, TGF-β1, and GAPDH in MG-63 Osteoblast-Like Cells

**Figure 3:**
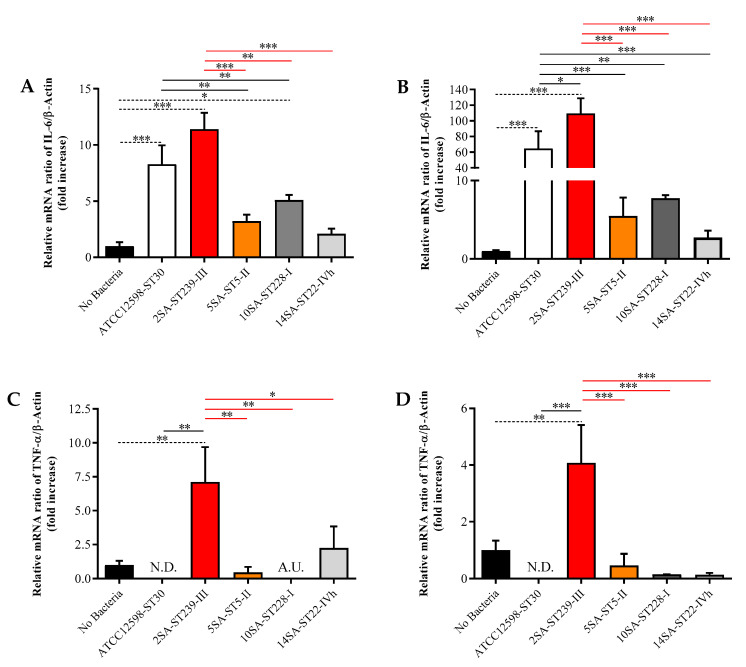
Gene expression of interleukin 6 (IL-6) (**A**,**B**) and tumor necrosis factor alpha (TNF-α) (**C**,**D**) in uninfected (No Bacteria) MG-63 osteoblast-like cells and in MG-63 cells infected at an MOI of 100:1 with five different *S. aureus* strains detected at 3 and 24 h p.i. N.D. = not detectable; A.U. = almost undetectable. The abundance of each mRNA is expressed relative to the abundance of β-actin-mRNA. Values are means ± SD of three independent experiments. Significant compariScheme 0. ** significantly different, *p* < 0.05;** significantly different, *p* < 0.01; *** significantly different, *p* < 0.001.

For the gene expression analysis, we compared the values obtained for each *S. aureus* strain to each other, normalizing them to the values obtained for the cells in the absence of bacteria, indicated as No Bacteria. Figure 3 shows the different mRNA expression levels of two well-known pro-inflammatory cytokines often simultaneously over-expressed and implicated in a wide variety of inflammatory processes [46,48,49,50], namely IL-6 and TNF-α, following infection for 3 and 24 h with the five different bacterial strains.

The ST30, ST239, and ST228 strains were able to significantly up-regulate the expression of IL-6 mRNA at 3 h p.i. compared with uninfected cells (*p* < 0.001 for ST30 and ST239; *p* < 0.05 for ST228), with the maximum effect observed when MG-63 cells were infected with ST239 (~11-fold increase) (Figure 3A). The increase due to ST239 was also significantly higher than that induced by ST5 (*p* < 0.001), ST228 (*p* < 0.01), and ST22 (*p* < 0.001). Regarding the reference strain, its induction led to a significant up-regulation of IL-6 mRNA expression compared with ST5 and ST228 (*p* < 0.01 for both). The prolongation of the infection time (up to 24 h) exacerbated the bacterial pro-inflammatory effects, especially in the case of ST239, whose IL-6 mRNA expression levels were significantly higher than those observed in cells in the absence of bacteria (*p* < 0.001) as well as for ST30 (*p* < 0.05) and the remaining MRSA strains (*p* < 0.001 vs. all of them) (Figure 3B). A different trend was observed for TNF-α induction. In fact, ST239 was the only strain able to significantly enhance TNF-α mRNA expression levels at both 3 h (Figure 3C; *p* < 0.01 vs. No Bacteria, ST30, ST5, or ST228; *p* < 0.05 vs. ST22) and 24 h (Figure 3D; *p* < 0.01 vs. No Bacteria and *p* < 0.001 vs. the other four strains at both time points) p.i.

**Figure 4:**
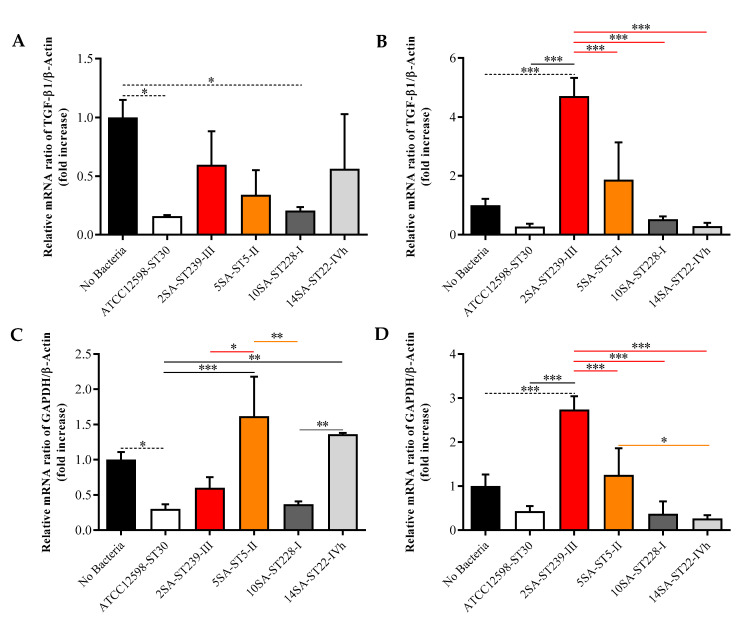
Gene expression of transforming growth factor beta 1 (TFG-β1) (**A**,**B**) and glycolytic enzyme glyceraldehyde 3-phosphate dehydrogenase (GAPDH) (**C**,**D**) in uninfected (No Bacteria) MG-63 osteoblast-like cells and in MG-63 cells infected at an MOI of 100:1 with five different *S. aureus* strains detected at 3 and 24 h. The abundance of each mRNA is expressed relative to the abundance of β-actin-mRNA. Values are means ± SD of three independent experiments. Significant comparisons between each experimental condition are indicated by lines. * Significantly different, *p* < 0.05; ** significantly different, *p* < 0.01; *** significantly different, *p* < 0.001.

Figure 4 shows the effects of the five bacterial strains on mRNA expression levels of TGF-β1, a cytokine overexpressed in osteomyelitis [51], and the glycolytic enzyme GAPDH, a metabolic marker [52].

Interestingly, the shorter infection time (3 h) led to a significant decrease in TGF-β1 mRNA expression levels for ST30 (*p* < 0.05) and ST228 (*p* < 0.05) compared with uninfected MG-63 cells (Figure 4A), while no significant differences were observed for the other three strains. Of note, at the prolonged infection time, the only clone able to significantly increase gene expression of TGF-β1 was ST239 (*p* < 0.001 vs. all other experimental conditions) (Figure 4B). In the case of GAPDH mRNA expression levels, none of the bacteria considered were able to induce up-regulation, and for the ST30 strain, the measured expression was even lower than that observed for uninfected cells (*p* < 0.05) (Figure 4C). Great variability was found among the different bacterial strains instead, with ST5 producing an up-regulation significantly higher than that observed for cells infected with ST30 (*p* < 0.001), ST239 (*p* < 0.05), or ST228 (*p* < 0.01). Some of the bacterial strains behaved very differently after prolonging the infection time (24 h), especially the ST239 strain. In fact, in the case of ST239-infected MG-63 cells, a significant induction of GAPDH mRNA was observed compared with all other experimental conditions (*p* < 0.001 vs. all) (Figure 4D). Surprisingly, the effect exerted by the ST5 strain, evident at 3 h p.i. compared with most of the other strains, was significant only when compared with ST22 (*p* < 0.05).

**Figure 5:**
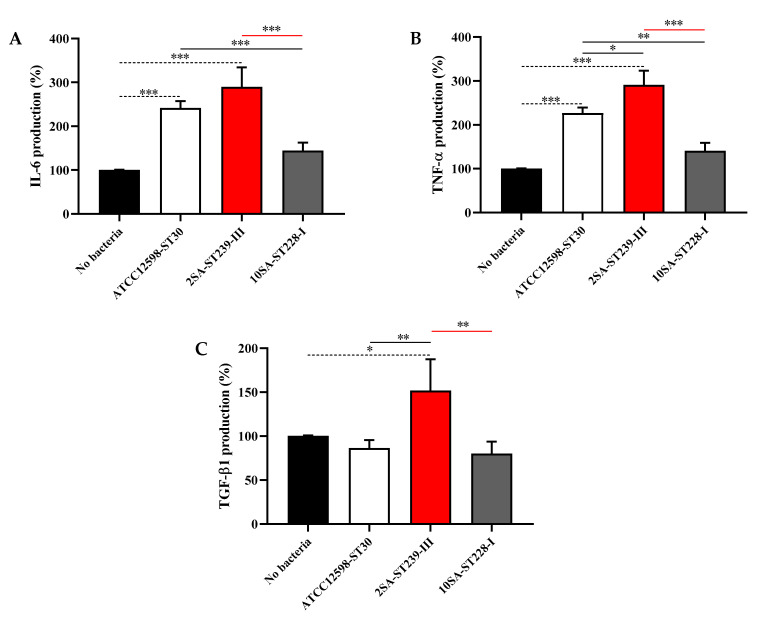
Modulation of interleukin 6 (IL-6), tumor necrosis factor alpha (TNF-α), and transforming growth factor beta 1 (TGF-β1) secretion by bacteria. Supernatants from uninfected (No Bacteria) MG-63 osteoblast-like cells and MG-63 cells infected at an MOI of 100:1 with three different *S. aureus* strains for 24 h were analyzed using a Custom Multi-Analyte ELISArray Kit. Each treatment was analyzed at least in triplicate. The production of each cytokine is expressed as the percent variation with respect to the production recorded in uninfected (control) cells. (**A**) IL-6, (**B**) TNF-α, and (**C**) TGF-β1. Values are means ± SD of three to four independent experiments. Significant comparisons between each experimental condition are indicated by lines. * Significantly different, *p* < 0.05; ** significantly different, *p* < 0.01; *** significantly different, *p* < 0.001.

To further confirm the results obtained by qRT-PCR, we performed additional tests using cell culture supernatants from uninfected MG-63 cells or cells infected at 24 h with ST30 (reference strain), ST239 (highest toxicity and internalization), or ST228 (lowest toxicity and internalization). The analysis of cytokines in cell supernatants indicated a significant up-regulation of the pro-inflammatory cytokine IL-6 induced by the presence of ST30 or ST239 compared with uninfected MG-63 cells (*p* < 0.001) (Figure 5A). *In contrast*, ST228 did not lead to any significant modulation of IL-6 release.

A quite comparable release profile was observed in the case of TNF-α (Figure 5B); in fact, both ST30 and ST239 treatments led to a significant increase in the secretion of TNF-α protein compared with uninfected MG-63 cells (*p* < 0.001). Of note, in this case, the presence of ST239 led to an increased secretion even compared with ST30 (*p* < 0.05). A different response to the considered bacteria was observed when measuring the release of TGF-β1. Neither ST30 nor ST228 were able to induce the release of the TGF-β1 cytokine compared with uninfected MG-63 cells. As expected, ST239 led to a significant increase in the release of TFG-β1 compared with all the other experimental conditions considered (*p* < 0.05 vs. No Bacteria and *p* < 0.01 vs. the other two strains) (Figure 5C).

### 3.4. The ST239 Strain Is the Least Effective in Modulating Nrf2 and HO-1 Gene Expression in MG-63 Osteoblast-Like Cells

**Figure 6:**
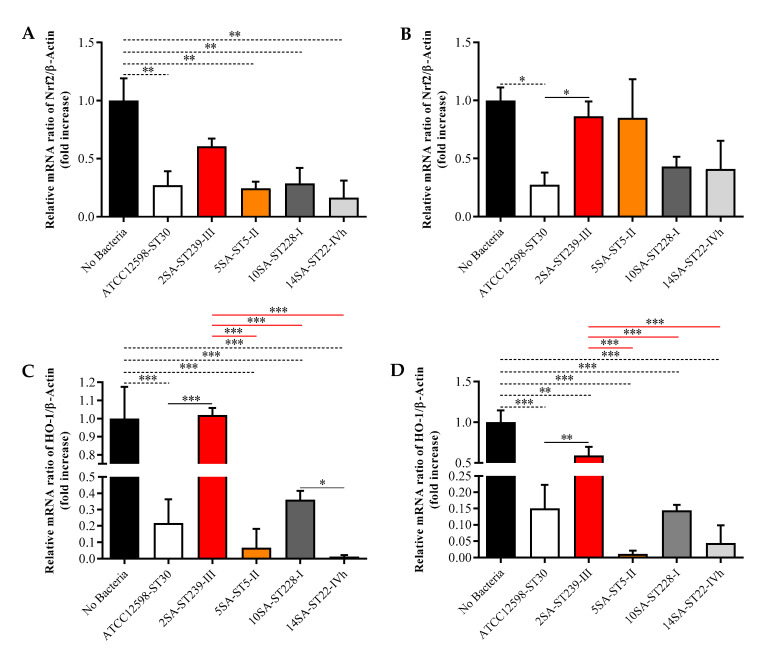
Gene expression of nuclear factor E2-related factor 2 (Nrf2) (**A**,**B**) and heme oxygenase 1 (HO-1) (**C**,**D**) in uninfected (No Bacteria) MG-63 osteoblast-like cells and in MG-63 cells infected at an MOI of 100:1 with five different *S. aureus* strains detected at 3 and 24 h. The abundance of each mRNA is expressed relative to the abundance of β-actin-mRNA. Values are means ± SD of three independent experiments. Significant comparisons between each experimental condition are indicated by lines. * Significantly different, *p* < 0.05; ** significantly different, *p* < 0.01; *** significantly different, *p* < 0.001.

Infecting MG-63 cells for 3 h led to a significant down-regulation of Nrf2 mRNA expression levels for all the strains considered (*p* < 0.01 for all of them) compared with uninfected cells, with the exception of ST239 (Figure 6A).

No significant differences were observed among the distinct bacterial strains at this time point. The prolongation of the time of infection (up to 24 h) *has evened out the differences between uninfected and infected cells, except in the case of the reference strain* ST30, which revealed Nrf2 mRNA expression levels significantly lower compared with both uninfected and ST239-infected cells (*p* < 0.05 for both) (Figure 6B). As observed in the case of Nrf2 at the shorter infection time, the treatment of MG-63 cells for 24 h led to a significant down-regulation of HO-1 mRNA expression levels (infected vs. uninfected) for all the strains considered (*p* < 0.001 for all of them), with the exception of ST239, whose values were comparable to those of control cells and significantly increased compared with all other strains (*p* < 0.001 vs. all) (Figure 6C). Very similar results were observed over the 24 h of infection, the only difference consisting of the down-regulation of the HO-1 mRNA expression values for the ST239 strain compared with uninfected cells (*p* < 0.01).

It is worth underlining that, despite the clear differences observed between the various strains, none of the bacteria considered were able to yield Nrf2 and HO-1 gene expression values higher than those measured in uninfected cells.

The above-mentioned results suggest that oxidative stress does not apparently contribute to bacteria-induced toxicity and pro-inflammatory phenomena. To further confirm this hypothesis, additional experiments were performed to measure the mRNA expression levels of iNOS and Nox-2. The activation of these two enzymes was closely linked to oxidative stress events [37]. As expected, infecting MG-63 cells with *S. aureus* strains did not induce any significant increase in iNOS and Nox2 mRNA expression levels (Supplementary Figure S2).

## 4. Discussion

Staphylococci, in particular *S. aureus*, are the predominant cause of bone infections worldwide [53]. *S. aureus*, one of the major human pathogens, is responsible for the altered homeostasis between bone cells during infections [28].

On the above basis, we used human MG-63 osteoblast-like cells infected with four different clinically isolated *S. aureus* strains and one strain of MSSA as a control with the aim to answer the following questions: (i) Is each strain’s ability to infect and persist in MG-63 osteoblast-like cells directly related to changes in cell viability? (ii) Do different genetic backgrounds lead to different pro-inflammatory and pro-oxidant responses in MG-63 osteoblast-like cells?

Our research group has recently used MG-63 cells as a tool to study host–pathogen interaction in a complex set of experiments including 16 different bacterial strains able to infect these cells to varying extents. In particular, the ability of MSSA ST30 and the two ST239 and ST22 MRSA to internalize and persist in MG-63 osteoblast-like cells was very similar (50.49% ± 0.69, 50.33% ± 1.19, and 45.37% ± 1.31, respectively), while the other MRSA clones, ST5 and ST228, behaved very differently in terms of internalization ability (27.59% ± 2.50 and 20.74% ± 1.04, respectively).

The answer to the first question was somewhat unexpected. In fact, even though ST30, ST239, and ST22 similarly demonstrated a higher internalization rate in MG-63 cells compared with ST5 and ST228, ST239 (at both 3 and 24 h) and ST5 (at 24 h) were the only strains that could significantly inhibit cell viability. In particular, despite having the same internalization rate as observed for the MSSA control strain, the ST239 clone was very toxic for MG-63 cells, whereas the ST228 clone, characterized by a lower internalization rate, exhibited a lower cytotoxic potential, comparable to the MSSA strain. Very interestingly, despite a similar decrease in cell viability induced by ST5 and ST239 at 24 h, the latter demonstrated greater adaptability to the hostile intracellular environment, being the only one that could maintain its numbers during the entire period of infection. ST239 is a well-studied and widespread clone presenting some genomic characteristic features [54] as well as virulence gene content [55]. A previous study on bacteremic patients demonstrated the development of a non-uniform and unique immune response against different staphylococcal proteins [56]; furthermore, a recent paper on MRSA ST239 identified a specific pattern of genes, confirming the differences among this clone and the others [57].

Specifically regarding the differences between MSSA and MRSA in their epidemiology and virulence, our findings are in line with different epidemiologic studies, including a meta-analysis in which an increased morbidity and/or mortality from MRSA compared with MSSA was shown [58,59,60,61].

The different behavior observed for these strains was also accompanied by a different modulation of inflammatory phenomena, metabolism, and antioxidant machinery.

In line with previously published data showing the inductive effects of *S. aureus* with regard to the expression of pro-inflammatory cytokines in different cell types [62,63] including osteoblasts [28], we found that the most persistent and at the same time most toxic strain, ST239, was able to strongly increase the expression levels of IL-6 and TNF-α, two cytokines often over-expressed during the development and progression of osteomyelitis [64,65,66,67] at both 3 and 24 h p.i. compared with other strains. It is worthwhile to describe more in detail the peculiarity of ST239′s pro-inflammatory activity. In fact, despite a comparable decrease in MG-63 cell viability observed for ST239 or ST5 24 h p.i., ST239 led to a very significant up-regulation of IL-6 and TNF-α. On the other hand, a completely different behavior (i.e., absence of induction) was observed for ST5 (values comparable to uninfected MG-63 osteoblast-like cells). This is relevant in the case of *S. aureus* infection; in fact, as recently suggested by Di Domenico et al., the production of pro-inflammatory cytokines can selectively promote *S. aureus* outgrowth and, interestingly, there is an interplay between host pro-inflammatory cytokines and bacterial biofilm production [68]. In particular, the pro-inflammatory cytokines produced during *S. aureus* infections can promote its outgrowth, subverting the composition of a healthy skin microbiome. In addition, biofilm production plays a relevant role in supporting chronic colonization, providing increased resistance to antimicrobial agents. Still on ST239, this strain was also the only one that could not modulate Nrf2 and its downstream gene HO-1 at both time points, showing that its pro-inflammatory activity is not linked to the modulation of the antioxidant machinery.

We then focused our attention on TGF-β1, a highly conserved anti-inflammatory cytokine, which acts as a key modulator of the microbiota and host immune cell cross-talk [69]. This cytokine was also associated in scar formation in osteomyelitis [51]. TGF-β1 plays a central role in immune suppression and repair after injury [70] and, most importantly, in the development and maintenance of bone-competent cells, such as osteoblasts and osteoclasts [71]. As has recently been demonstrated in vivo by Wang et al. employing a rat model mimicking the biological development of osteomyelitis, the expression of TGF-β1 gene and its receptors (TβRI and TβRII) is strongly up-regulated [51]. TβRI and TβRII are also expressed by the MG-63 osteoblast-like cells used in the present study [72]. TGF-β1 is a stimulator of type I collagen production [73], which is connected to the hypertrophic scarring of the soft tissue surrounding the infected bone in osteomyelitis [74]. Accordingly, ST239, the more active clone in terms of invasiveness, toxicity, and inflammation, was the only one able to significantly enhance TGF-β1 gene and protein expression levels 24 h p.i. compared with untreated cells and the other four strains. In a recent review, Lamora et al. underlined that TGF-β1 cytokine production plays a pivotal role in osteosarcoma progression through its pro-metastatic effects [75]. This indication, considering the origin of the cell model used in this study, MG-63 cells (a line derived from osteosarcoma), may shed a different light on our results. Nevertheless, the new data discussed in this paper suggest a potential role of TGF-β1 in the pathophysiology of osteomyelitis. We hypothesize that TGF-β1 may initially increase in response to bacteria-related inflammation and to augmented secretion of pro-inflammatory cytokines, thus gaining a pathogenic role promoting fibrosis in osteomyelitis, as demonstrated in vivo.

As has been seen for TGF-β1, from a metabolic point of view, the presence of ST239 inside MG-63 osteoblast-like cells was the only condition to significantly enhance the metabolic status of cells, as evaluated through measurement of GAPDH mRNA expression levels [52]. It is noteworthy that this metabolic enhancement was, as observed for TGF-β1, clearly evident only at 24 h p.i. The presence of GAPDH has been connected to virulence and to the adhesion of several pathogenic microorganisms [76], while its enhanced gene expression has been reported to participate in cell death phenomena [77]; both of these findings support the highest toxicity on MG-63 cells observed under our experimental conditions.

With the aim of verifying whether oxidative stress and the activation of antioxidant machinery were connected to the changes observed in MG-63 cells following bacterial infection, the expression levels of Nrf2 and HO-1 were measured under each of our experimental conditions. None of the bacteria, including ST239, were able to provide Nrf2 and HO-1 gene expression values higher than those measured in uninfected cells; indeed, MRSA strains significantly down-regulated Nrf2 at the shorter time point and HO-1 at both 3 and 24 h p.i. Of note, ST239 was the least effective strain in modulating Nrf2 and HO-1 gene expression in MG-63 osteoblast-like cells. The minor role played by oxidative stress was also emphasized by the absence of modulation of iNOS and Nox2 gene expression at all the conditions considered. On the basis of these data, we hypothesize the following: (1) an infection time longer than 24 h is needed for the activation of both pro-oxidant enzymes and antioxidant machinery; (2) staphylococci are able to negatively modulate endogenous and exogenous oxidative and nitrosative stress [78].

## 5. Conclusions

Overall, our data suggest an extremely heterogeneous response of MG-63 cells infected with bacteria belonging to the same species. This seems to suggest that a “general” approach should be replaced by a “clonal approach” for the treatment of *S. aureus* infections, paying particular attention to the importance of strain-specific behaviors related to specific hosts/infections. Our findings pave the way for potential alternative therapeutic strategies against *Staphylococcus*-induced damage that consider the use of molecules able to counteract the activity of pro-inflammatory cytokines, e.g., selective cytokine signaling inhibitors (IL-6, TNF-α, and TGF-β1) or, alternatively, neutralizing antibodies or recombinant soluble receptors.

## Figures and Tables

**Table 1:** Phenotypical and molecular characteristics of the five different *S. aureus* strains included in the study.

**LAB CODE**	**ST-SCC*mec*-spa Type**	**Source**	**FOX**	**CN**	**DA**	**E**	**CIP**	**TE**	**SXT**	**K**	**RD**	**BPR**
ATCC-12598	30-MSSA-III-t976	-	-	-	-	-	-	-	-	-	-	-
2SA	239-III-t037	wound	R	R	Ri	R	R	R	R	R	2	2
5SA	5-II-t2154	blood	R	R	S	R	R	R	S	R	>32	2
10SA	228-I-t041	blood	R	R	R	R	R	S	S	R	>32	2
14SA	22-IVh-t032	blood	R	S	Ri	R	R	S	S	S	0.008	1
**LAB CODE**	**ST-SCC*mec*-spa type**	**Source**	**DAL**	**CPT**	**LNZ**	**DPT**	**TGC**	**FU**	**VA**	**TC**	**GRD**	
ATCC-12598	30-MSSA-III-t976	-	-	-	-	-	-	-	-	-	-	
2SA	239-III-t037	wound	0.125	2	2	0.5	0.25	>256	1	2	VSSA	
5SA	5-II-t2154	blood	0.012	4	32	0.5	0.5	>256	1	0.5	hVISA	
10SA	228-I-t041	blood	0.125	1	8	1	0.125	0.125	1	1	VSSA	
14SA	22-IVh-t032	blood	0.064	1	1	1	0.125	0.064	0.5	0.25	VSSA	

FOX: cefoxitin; CN: gentamicin; DA: clindamycin; E: erythromycin; CIP: ciprofloxacin; TE: tetracycline; SXT: trimethoprim/sulfamethoxazole; K: kanamycin; RD: rifampin; BPR: ceftobiprole; DAL: dalbavancin; CPT: ceftaroline; LNZ: linezolid; DPT: daptomycin; TGC: tigecycline; FU: fusidic acid; VA: vancomycin; TC: teicoplanin; GRD: MIC test strip glycopeptide-resistance detection; Ri: inducible clindamycin resistance; clone characterization by means of the following: ST—sequence type; SCCmec—Staphylococcal cassette chromosome mec; spa type—staphylococcal protein A.

**Table 2:** List of primers used for quantitative real-time PCR (qRT-PCR).

Official Name ^#^	Official Symbol	Alternative Titles/Symbols	Detected Transcript	Amplicon Length	Cat. No. ^§^
nitric oxide synthase 2, inducible	NOS2	NOS; INOS; NOS2A; HEP-NOS	NM_000625 NM_153292	92 bp	QT00068740
cytochrome b-245 beta chain	CYBB	CGD; NOX2; IMD34; AMCBX2; GP91-1; GP91PHOX; p91-PHOX; GP91-PHOX	NM_000397	124 bp	QT00029533
transforming growth factor beta 1	TGFB1	CED; LAP; DPD1; TGFB; IBDIMDE; TGFbeta; TGF-beta1	NM_000660	108 bp	QT00000728
interleukin 6	IL6	CDF; HGF; HSF; BSF2; IL-6; BSF-2; IFNB2; IFN-beta-2	NM_000600 XM_005249745	107 bp	QT00083720
tumor necrosis factor	TNF	DIF; TNFA; TNFSF2; TNLG1F; TNF-alpha	NM_000594	98 bp	QT00029162
glyceraldehyde-3-phosphate dehydrogenase	GAPDH	G3PD; GAPD; HEL-S-162eP	NM_001256799 NM_002046 NM_001289745 NM_001289746	95 bp	QT00079247
nuclear factor, erythroid 2 like 2	NFE2L2	NRF2; HEBP1; Nrf-2; IMDDHH	NM_006164	153 bp	QT00027384
heme oxygenase 1	HMOX1	HO-1; HSP32; HMOX1D; bK286B10	NM_002133	99 bp	QT00092645
actin beta	ACTB	BRWS1; PS1TP5BP1	NM_001101	146 bp	QT00095431

^#^https://www.ncbi.nlm.nih.gov/gene/, ^§^https://www.qiagen.com/it/shop/pcr/real-time-pcr-enzymes-and-kits/two-step-qrt-pcr/quantitect-primer-assays/.

## Supplementary Materials

The following are available online at https://www.mdpi.com/2218-273X/11/1/72/s1, Figure S1: Changes in absorbance values measured at 569 nm at five different concentrations of free bacteria; Figure S2: Gene expression of NADPH oxidase 2 (Nox-2) (**A**,**B**) and inducible nitric oxide synthase (iNOS) (**C**,**D**) in uninfected (No Bacteria) MG-63 osteoblast-like cells and in MG-63 cells infected at an MOI of 100:1 with five different *S. aureus* strains detected at 3 and 24 h; Table S1: Additional statistics regarding the comparison of spots counting measured under all our experimental conditions.
