# Supplementary material for: Different Modulatory Effects of Four Methicillin-Resistant Staphylococcus aureus Clones on MG-63 Osteoblast-Like Cells
Source: Biomolecules. 2021 Jan 7;11(1):72. doi: 10.3390/biom11010072 (PMC7825699; doi:10.3390/biom11010072)
Supplement: Supplementary file 1 [file biomolecules-11-00072-s001.pdf]

Supplementary Materials

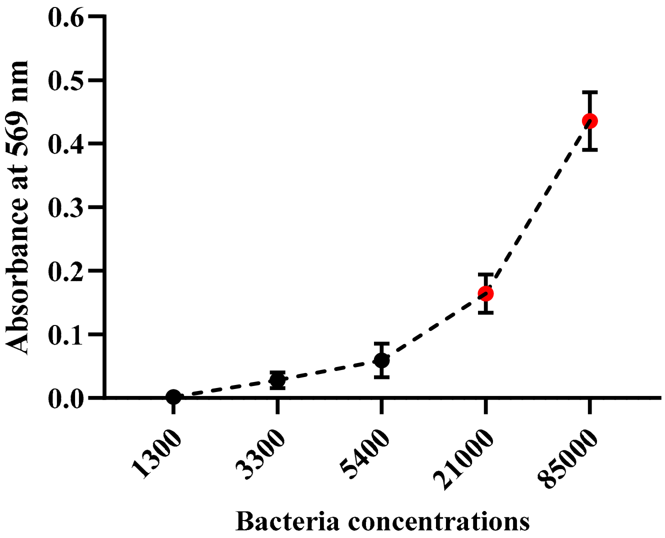

Supplementary Figure S1. Changes in absorbance values measured at 569 nm at five different concentrations of free bacteria. The red circles indicate the concentrations at which the bacteria metabolize a significant amount of MTT salt. Values are reported as means ± SD of three measurements.

Supplementary Table S1. Additional statistics regarding the comparison of spots counting measured under all our experimental conditions. Only percentages belonging to the same spot category were compared.

Bonferroni Multiple Comparisons Test	*p*-value
ST30:0 spots vs. ST239:0 spots	>0.9999
ST30:0 spots vs. ST5:0 spots	<0.0001
ST30:0 spots vs. ST228:0 spots	<0.0001
ST30:0 spots vs. ST22:0 spots	>0.9999
ST30:1–5 spots vs. ST239:1–5 spots	>0.9999
ST30:1–5 spots vs. ST5:1–5 spots	<0.0001
ST30:1–5 spots vs. ST228:1–5 spots	<0.0001
ST30:1–5 spots vs. ST22:1–5 spots	>0.9999
ST30:>5 spots vs. ST239:>5 spots	>0.9999
ST30:>5 spots vs. ST5:>5 spots	>0.9999
ST30:>5 spots vs. ST228:>5 spots	>0.9999
ST30:>5 spots vs. ST22:>5 spots	>0.9999
ST239:0 spots vs. ST5:0 spots	<0.0001
ST239:0 spots vs. ST228:0 spots	<0.0001
ST239:0 spots vs. ST22:0 spots	>0.9999
ST239:1–5 spots vs. ST5:1–5 spots	<0.0001
ST239:1–5 spots vs. ST228:1–5 spots	<0.0001
ST239:1–5 spots vs. ST22:1–5 spots	>0.9999
ST239:>5 spots vs. ST5:>5 spots	>0.9999
ST239:>5 spots vs. ST228:>5 spots	>0.9999
ST239:>5 spots vs. ST22:>5 spots	>0.9999
ST5:0 spots vs. ST228:0 spots	>0.9999
ST5:0 spots vs. ST22:0 spots	<0.0001
ST5:1–5 spots vs. ST228:1–5 spots	>0.9999
ST5:1–5 spots vs. ST22:1–5 spots	<0.0001
ST5:>5 spots vs. ST228:>5 spots	>0.9999
ST5:>5 spots vs. ST22:>5 spots	>0.9999
ST228:0 spots vs. ST22:0 spots	<0.0001
ST228:1–5 spots vs. ST22:1–5 spots	<0.0001
ST228:>5 spots vs. ST22:>5 spots	>0.9999

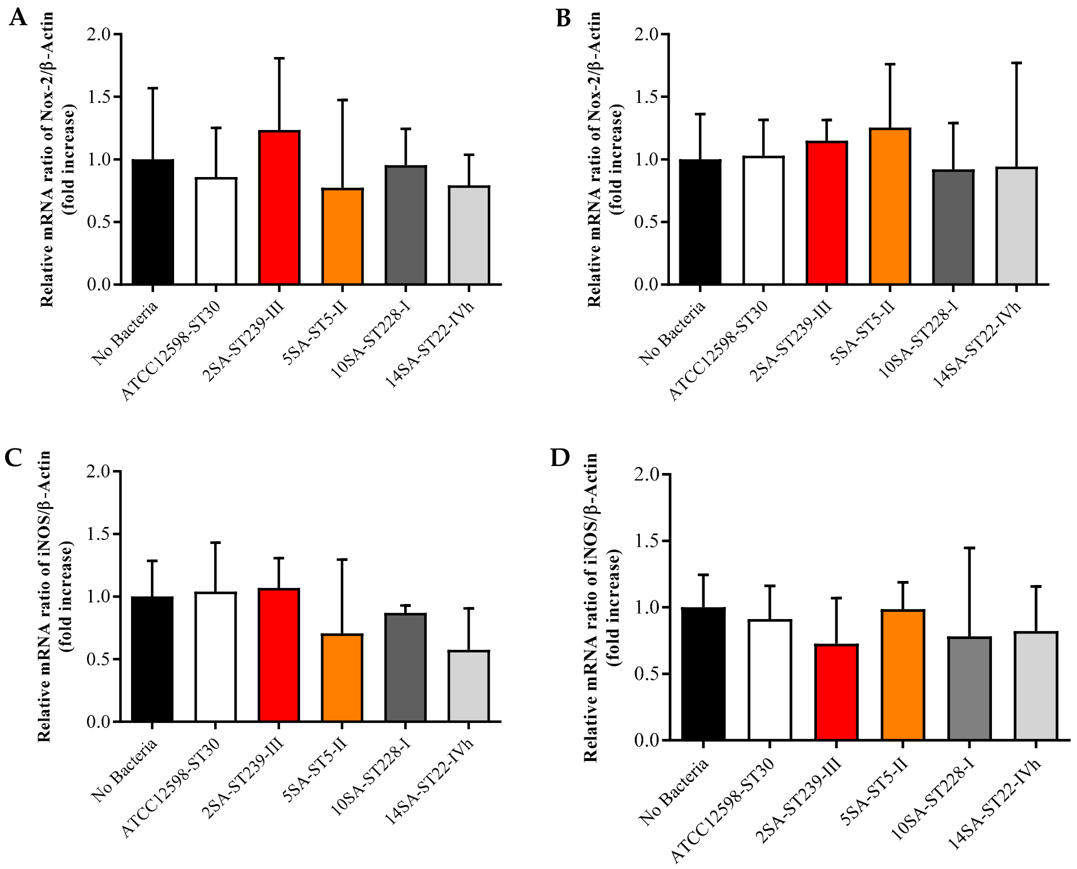

Supplementary Figure S2. Gene expression of Nox-2 (**A**,**B**) and iNOS (**C**,**D**) in uninfected (no bacteria) MG-63 osteoblast-like cells and in MG-63 cells infected at a MOI of 100:1 with five different *S. aureus* strains detected at 3 and 24 h. The abundance of each mRNA is expressed relative to the abundance of β-actin-mRNA. Values are means ± SD of three independent experiments.
